# Supplementary figures and images for: α-parvin is required for epidermal morphogenesis, hair follicle development and basal keratinocyte polarity
Source: PLoS One. 2020 Mar 12;15(3):e0230380. doi: 10.1371/journal.pone.0230380 (PMC7067437; doi:10.1371/journal.pone.0230380)

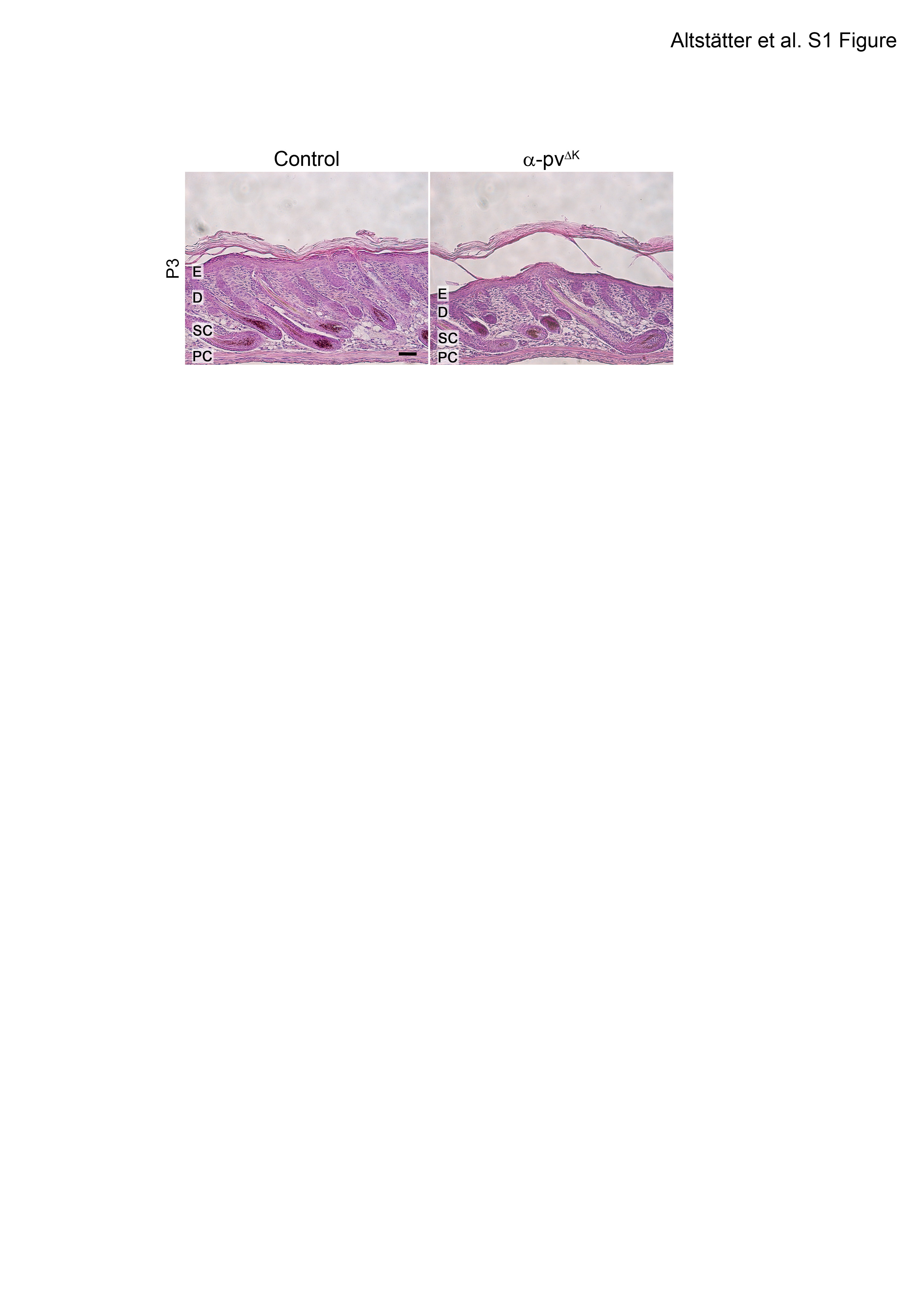

Supplement: S1 Fig — Hematoxylin-eosin staining of back skin section of 3-days-old control and α-pvΔK mice did not reveal any significant differences between control and α-pvΔK mice. E: epidermis; D: dermis; SC: subcutis; PC: panniculus carnosum. Scale bar: 200 μm. (TIF) [file pone.0230380.s001.tif]

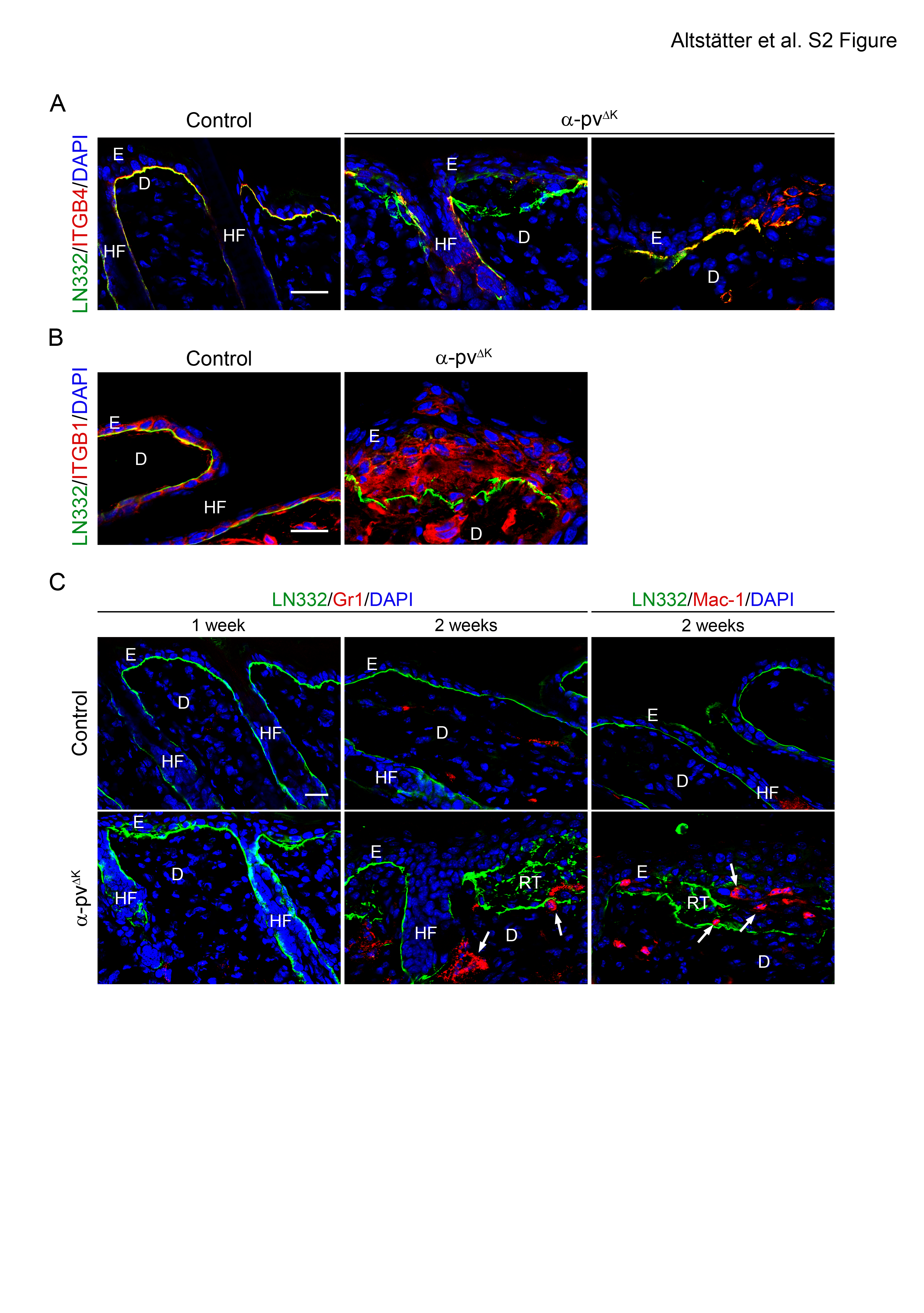

Supplement: S2 Fig — Double-fluorescent labeling for (A) LN332 and β4-integrin, and (B) LN332 and β1-integrin of control and α-pvΔK skin sections. Nuclei were visualized with DAPI. Scale bar: 20 μm. (C) Double-fluorescent labeling for LN332 and Gr1, and LN332 and Mac-1 of control and α-pvΔK skin sections. Nuclei were visualized with DAPI. Scale bar: 20 μm. E: epidermis, D: dermis, HF: hair follicle and RT: repair tissue. (TIF) [file pone.0230380.s002.tif]

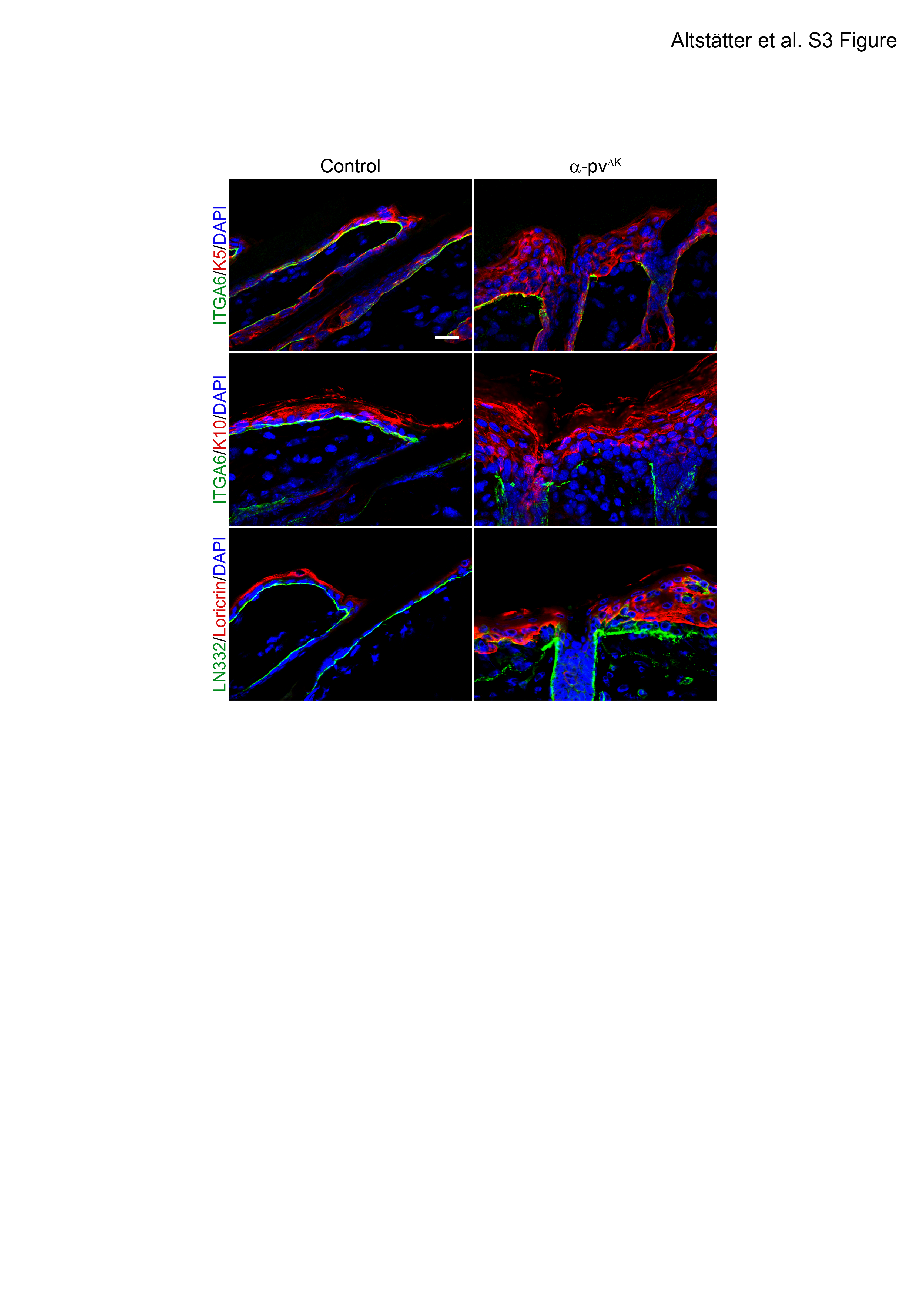

Supplement: S3 Fig — Double-fluorescent labeling for keratin-5 and α6-integrin, keratin-10 and α6-integrin, and loricrin and α6-integrin of control and α-pv-null skin sections. Nuclei were visualized with DAPI. Scale bar: 20 μm. (TIF) [file pone.0230380.s003.tif]

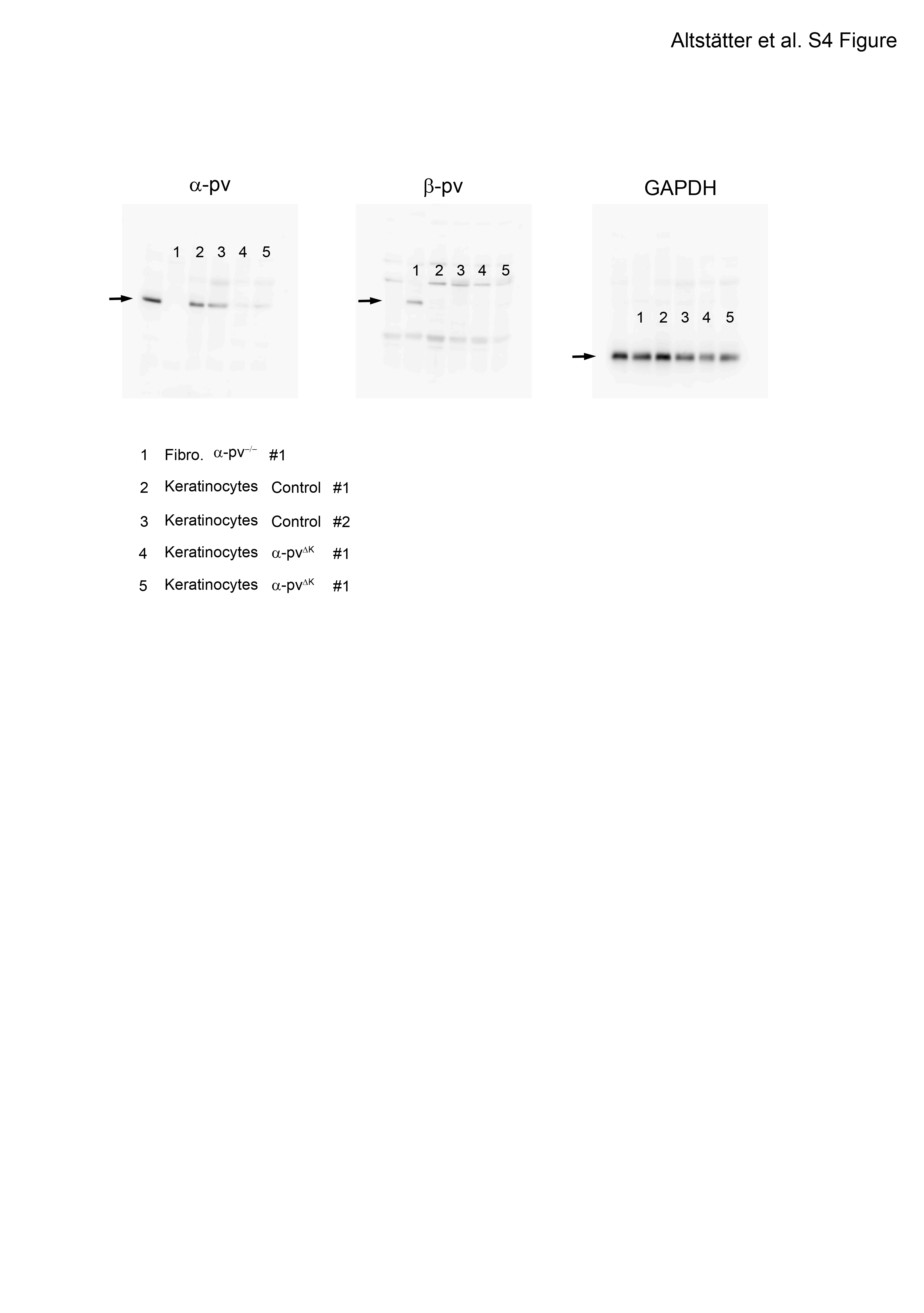

Supplement: S1 Raw images — (TIF) [file pone.0230380.s004.tif]
