# Supplementary material for: α-parvin is required for epidermal morphogenesis, hair follicle development and basal keratinocyte polarity
Source: PLoS One. 2020 Mar 12;15(3):e0230380. doi: 10.1371/journal.pone.0230380 (PMC7067437; doi:10.1371/journal.pone.0230380)
Supplement: Attachment — Submitted filename: renamed_9d784.docx [file pone.0230380.s005.docx]

**PONE-D-20-01092**

α-parvin is required for epidermal morphogenesis, hair follicle development and basal keratinocyte polarity

The answers from the authors are in red.

**1. Journal Requirements**

1. Please ensure that your manuscript meets PLOS ONE's style requirements, including those for file naming. The PLOS ONE style templates can be found at <http://www.plosone.org/attachments/PLOSOne_formatting_sample_main_body.pdf> and <http://www.plosone.org/attachments/PLOSOne_formatting_sample_title_authors_affiliations.pdf>

We have reformatted the manuscript to meet the style requirements of PLOS ONE.

2. We note that one or more of the authors are employed by a commercial company: Roche Diagnostics GmbH.

Please provide an amended Funding Statement declaring this commercial affiliation, as well as a statement regarding the Role of Funders in your study. If the funding organization did not play a role in the study design, data collection and analysis, decision to publish, or preparation of the manuscript and only provided financial support in the form of authors' salaries and/or research materials, please review your statements relating to the author contributions, and ensure you have specifically and accurately indicated the role(s) that these authors had in your study. You can update author roles in the Author Contributions section of the online submission form.

Please also include the following statement within your amended Funding Statement.

“The funder provided support in the form of salaries for authors [insert relevant initials], but did not have any additional role in the study design, data collection and analysis, decision to publish, or preparation of the manuscript. The specific roles of these authors are articulated in the ‘author contributions’ section.”

If your commercial affiliation did play a role in your study, please state and explain this role within your updated Funding Statement.

Please also provide an updated Competing Interests Statement declaring this commercial affiliation along with any other relevant declarations relating to employment, consultancy, patents, products in development, or marketed products, etc.

Within your Competing Interests Statement, please confirm that this commercial affiliation does not alter your adherence to all PLOS ONE policies on sharing data and materials by including the following statement: "This does not alter our adherence to PLOS ONE policies on sharing data and materials.” (as detailed online in our guide for authors http://journals.plos.org/plosone/s/competing-interests) . If this adherence statement is not accurate and there are restrictions on sharing of data and/or materials, please state these. Please note that we cannot proceed with consideration of your article until this information has been declared.

During the entire duration of the work, Dr. Johannes Altstätter was employed exclusively at the Max Planck Institute, which paid his salary as a doctoral student. Moreover, the work has been entirely financed by the Max Planck Society. Therefore, to avoid any confusion, we have removed Dr. Altstätter's current affiliation with Roche Diagnostics GmbH from the manuscript.

Please include both an updated Funding Statement and Competing Interests Statement in your cover letter. We will change the online submission form on your behalf.

Funding Statement: the work was funded by the Max Planck Society.

Competing Interests Statement: the authors state no conflict of interest.

3. PLOS ONE now requires that authors provide the original uncropped and unadjusted images underlying all blot or gel results reported in a submission’s figures or Supporting Information files. This policy and the journal’s other requirements for blot/gel reporting and figure preparation are described in detail at https://journals.plos.org/plosone/s/figures#loc-blot-and-gel-reporting-requirements and https://journals.plos.org/plosone/s/figures#loc-preparing-figures-from-image-files. When you submit your revised manuscript, please ensure that your figures adhere fully to these guidelines and provide the original underlying images for all blot or gel data reported in your submission. See the following link for instructions on providing the original image data: https://journals.plos.org/plosone/s/figures#loc-original-images-for-blots-and-gels.

The original uncropped and unadjusted images of all blots are provided as supporting information (S1_raw_images).

4. We note that you have included the phrase “data not shown” in your manuscript. Unfortunately, this does not meet our data sharing requirements. PLOS does not permit references to inaccessible data. We require that authors provide all relevant data within the paper, Supporting Information files, or in an acceptable, public repository. Please add a citation to support this phrase or upload the data that corresponds with these findings to a stable repository (such as Figshare or Dryad) and provide and URLs, DOIs, or accession numbers that may be used to access these data. Or, if the data are not a core part of the research being presented in your study, we ask that you remove the phrase that refers to these data.

We provide all relevant data as supplementary information (S1 Fig).

**2. Review Comments to the Author**

We would like to thank all reviewers for their time, effort and valuable suggestions, which are greatly appreciated and have enabled us to improve the manuscript further.

**Reviewer #1:** This is the first report of an epidermis-specific inactivation of α-parvin. The claim in the title that α-parvin is required for epidermal morphogenesis, hair follicle development and keratinocyte polarity are justified based on the morphological images presented in the manuscript. In contrast, claims that inactivation of α-parvin results in weakened keratinocyte-BM adhesion and rupture of the BM are not supported by the data.

This is an important observation, thank you for the comment.

We have modified the abstract, the results and the discussion to avoid any misunderstandings.

The revised abstract now reads “*Inactivation of the murine α-pv gene in basal keratinocytes results in keratinocyte-BM detachment, epidermal thickening, ectopic keratinocyte proliferation and altered actin cytoskeleton polarization*” and the revised discussion that now reads “*Deletion of the α-pv gene in keratinocytes in mice has three main consequences: 1) keratinocyte-BM detachment resulting in subepidermal blisters and distorted BM organization…*”

Major problems:

1. Most of the pictures are at high magnification. Consequently only a small portion of the section is shown while an overview at low magnification would allow a much better evaluation of skin defects. Observation at high magnification may have misled the authors. Indeed, there are blisters in Fig.2a, 2 weeks; and repair tissue filling the blister cavity in Fig.2a, 4 weeks, just below the asterisk; probably in Fig.2a, 8 weeks between the epidermis and the melanin deposits; and in Fig. 3b; in Fig. 4a, left photo of α-PVΔK between epidermis and the LN332-containing BM along the cavity floor; in Fig. 4b, photo of α-PVΔK between epidermis and the LN332-containing BM along the cavity floor (here the repair tissue is staining positive for beta 1 integrin as it is typical for repair tissue during wound healing); in Fig. 4d, the two pictures corresponding to α-PVΔK 2 weeks staining positive for LN332, Gr1 and Mac-1 (positive staining with Gr1 and Mac-1 is typical of the inflammatory activation during wound healing).

Several photos are centered on this repair tissue (b1 integrins, Mac-1, Gr1) and not on the epidermis as claimed by the authors.

Thank you for this comment. The reviewer is correct and in many images it is possible to observe the blisters filled the repair tissue. To avoid any misunderstandings, we have depicted the repair tissue areas in the revised figure 2 and figure 4. Please also see point 3.

2. Figure 1a,b aims to show parvin expression in skin. The origin of the rabbit antibody is not mentioned in Material and Methods.

We apologize for this shortcoming. The anti-alpha-parvin rabbit antibody was provided by Dr. Reinhard Fässler. We now provide this information in the revised material and methods section. We have also incorporated a reference into the material and methods in the revised manuscript (ref.17)

Very frequently rabbit antiserum contains antibodies against rabbit keratins. Staining of the paraffin section looks as a basal keratin staining, i.e. without polarization. The authors should show strictly parallel staining of control and KO skin side by side to be convincing.

Thank you for this suggestion. We have performed immunostaining of skin samples from 2 weeks-old α-pv^ΔK^ mice with the anti-alpha-parvin antibody. The analysis revealed a strong reduction in the α-pv signal in α-pv^ΔK^ mice when compared to control mice. These results are displayed in revised figure 1A and mentioned in revised results sections.

Where is parvin in the whole-mount?

We apologize for not been clear in this point. As showed in figure 1A and mentioned in the results part, α-pv is expressed in basal keratinocytes of interfollicular epidermis and ORS of the HFs. To make this point clear, this is now also mentioned in the revised figure legend.

Are the keratinocytes used for the western blot cultivated or freshly isolated?

We apologize for this shortcoming. The keratinocytes used for the western blot were freshly isolated. This information is now mentioned in the revised results section.

And what about the fibro? I guess it stands for fibroblasts which are not mentioned in the legend or in Material and Methods.

The reviewer is correct, “fibro” stands for fibroblast. This information is mentioned in the revised figure legend.

3. Figure 2 shows blisters at 2 weeks and not later. Is that correct? If yes what happen to the blisters at time point later than 2 weeks? Where are the blisters occurring? subepidermal or intraepidermal? and how much of the dermal-epidermal zone is affected.

We apologize for not been clear in this point. The subepidermal microblisters are also seen at 4 and 8 weeks. In the original figure 2A, the microblisters at 8 weeks were marked with a black arrow. In the revised figure 2A, the microblisters filled with repair tissue at 4 weeks are pointed with a arrow.

Overview should be provided. Explicit a, b and c separately in the legend.

We believe that the overview images provided in figure 4A and the high magnification images provided in figures 4B and 4C adequately illustrate the skin defects in α-pv^ΔK^ mice.

Details related to quantification of HFs are totally missing in the Legend or in Material and Methods (how many mice, how many sections per mice, which software, etc…).

We apologize for this shortcoming. We provided all necessary information in revised legend of figure 2.

4. Figure 3b, Figure 4a,b,d and Figure 5: For the same reasons as above, overview should be provided. As mentioned above, some pictures are centered to the repair tissue filling the blister cavity while others are centered on the epidermis outside the damaged areas where the dermal-epidermal junction appears to be intact. This is very confusing.

Thank you for this suggestion. We provide overview images in revised figure 3B and new S2 and S3 figures. Moreover, to avoid any misinterpretations of the results, we have depicted the repair tissue areas in the revised figure 4.

5. The blister level is not within the basement membrane plane since in Fig. 4 the electron micrographs of the knockout animals shown in c, there is a distinct basal lamina in area B. In addition for α-PVΔK skin, there is good positive staining of LN332 in the floor of the blister/filled blister cavity in Fig. 3b, Fig. 4a,b Fig. 4d5.

Thank you for this comment. The skin of α-pv^ΔK^ mice showed extensive areas with a discontinuous and disordered BM, where LN332 was also detected in the dermal region below the BM plane. As we mention in the results section we focus our EM analysis in areas with distorted BM.

In conclusion, better illustrations should be provided and interpretation of the results needs a careful re-examination.

**Reviewer #2:** This is a nice manuscript describing the parvin knockout phenotype in skin. The analysis was interesting and well documented. The authors show that epidermal morphogenesis and hair follicle formation are critically dependent upon this key Integrin signalling component. I recommend publication.

We thank the reviewer for recommending the publication of our manuscript without further modifications.
